# Supplementary material for: Chromothripsis during telomere crisis is independent of NHEJ, and consistent with a replicative origin
Source: Genome Res. 2019 May;29(5):737–49. doi: 10.1101/gr.240705.118 (PMC6499312; doi:10.1101/gr.240705.118)
Supplement: Supplemental Material [file supp_gr.240705.118_Supplemental_file_1.zip › contigs/annotated_contigs/DB95/contig.2.DB95_length_796_mean_cov_5.27638190955.docx]

**DB95_length_796_mean_cov_5.27638190955**

```
TAATCTCTAATTTATTTTAGATCATTTTGAAACACTGTGGTTTCAGTTTTTATCTGCTTTGTGGACTTATCCTGCAGGTGTAATCTCCT
 >chr13:110506517-110506965 + E=1e-254
TATCTGCAGGAACAATATTTTGTTCCTTATTCCTTTCCTTGGAGTGACTTTATATGGACTTCTGCAACCCTTTTCTGTTACTCATTTTT

ACCTGAGATTCACTTTCCTGAATTTTTAGGAGGGAGAGATGAGTCTAAAGAGTGTTTTCAGCTCCAGGCTCTGGACCTCTCTCTGGAGT

TGTTTTCCTGAAACCATTAAGTGTGGGGCTGGTCCTTGTTCACCCACCCAGGATCCCTGTCCCTCCCTCTCCCCCCTTCTCTCTCCTCC

CTACCATCTCTATCCTGCTCTTTTGTATTCTCTTCCCACCAGCTTCTCCTCTTTGTGGCATTGAGTCCTGGAGGGAGCTTGGCTTG|TT
                                                                                       >
GGTT|ATTTTTGCTGTTTTTGCCTGAGCTTTTGGTGTGATTAAAAAAAAAAGCATTGCTAAGGCCAATATCAAGGAGTTTTCTTCTTAT
chr13:110691057-110691413 + E=3e-181
GTTTTCTTCCAGGAGTTTTAGTTTCAAGTGTTCTGTTTAGGTCTTTAATTCACTTTGAGCTGATTTTTGTGTATGGTGTGAGACAAGAG

CCCATTGTCATTCCTTTGAATGTGTATATATAGTTTTCCTGCACAATTTATTGAAGAAACCATCCTTTCTTCACTAACATATGCAAATC

AATCAGTGTGGCATACCACATAACCTCATGGTCATCTCAGTGGATGGAGAAAAAGCATTTGACACAGTTCAACATCCTTTCATGAT
```
